# Oligometastatic Prostate Cancer: Is there a Role for Surgery? A Narrative Review

**DOI:** 10.5152/tud.2022.22064

**Published:** 2022-05-01

**Authors:** Eugenio Martorana, Morgan Bruschi, Pietro Scialpi, Riccardo Grisanti, Michele Scialpi

**Affiliations:** 1 Department of Urology, Nuovo Ospedale Civile di Sassuolo, Modena, Italy; 2 Division of Urology, Portogruaro Hospital, Venice, Italy; 3 Division of Diagnostic Immaging, Department of Medicine and Surgery, S. Maria della Misericordia Hospital, Perugia University, Italy

**Keywords:** Oligometastatic Prostate Cancer, cytoreductive radical prostatectomy, safety, feasibility, oncological outcomes.

## Abstract

Oligometastatic prostate cancer is commonly considered a transition between high metastatic and localized disease and includes a large spectrum of conditions with a polymorphic clinical behavior. The current management of these patients contemplates systemic therapy (i.e., androgen-deprivation drugs, chemotherapeutic drugs, or both treatments administered simultaneously) which have been shown to improve survival. Radiotherapy has also been introduced, into a multimodal setting, among the therapeutic treatments for patients who are defined as oligometastatic prostate cancer according to Chemohormonal Therapy Versus Androgen Ablation Randomized Trial for Extensive Disease in Prostate Cancer (CHAARTED) criteria. The role of surgical debulking in patients with oligometastatic prostate cancer has always been considered impracticable, both for a marginal therapeutic role and for the greater risk of sequelae and/or complications related to the procedure itself. Several authors have demonstrated some mechanisms by which the persistence of the primary tumor can facilitate the clinical progression of the disease itself and promote carcinogenesis, differentiation, migration, and angiogenesis in prostate cancer. From these studies emerges the hypothesis of a possible therapeutic advantage in oncological terms also for cytoreductive radical prostatectomy, in a multimodal therapy setting, compared to systemic therapy alone. The present review summarizes the main knowledge regarding the safety, feasibility, and oncological outcomes of cytoreductive radical prostatectomy in oligometastatic prostate cancer patients.

**Main Points**

- Oligometastatic prostate cancer is considered a transition between high metastatic and localized disease and includes a large spectrum of conditions with a polymorphic clinical behavior.
- Some cell signals of primary tumor can facilitate the disease progression by promoting carcinogenesis, differentiation, migration, and angiogenesis.
- Cytoreductive radical prostatectomy (CRP) is safe, feasible, and well-tolerated in a well-selected group of patients and, despite its challenging execution, it is associated with a reduction of pelvic complications related to local disease progression.
- Oligometastatic prostate cancer patients may receive deep and durable oncological benefits from CRP within a multimodality setting.

## Introduction

Prostate cancer is the second most widespread cancer diagnosed in the United States, the third for cancer death with about 248 530 new cases and about 34 130 deaths in 2021.^1^

In 2018, about 20% of the 1.3 million worldwide with PCa diagnosed had metastatic disease.^2^

Oligometastatic prostate cancer (OMPC) is commonly defined by the presence of 5 or fewer metastatic sites on usual staging imaging. It is considered a transition between high metastatic and organ-confined disease and includes a large spectrum of conditions with a polymorphic clinical behavior.^3^

The current management of these patients contemplates systemic therapy (i.e., androgen-deprivation drugs, chemotherapeutic drugs, or both treatments administered simultaneously) which have been shown to improve survival. Radiotherapy has also been introduced, into a multimodal setting, among the therapeutic treatments of patients defined as OMPC according to CHAARTED criteria.^4^

The role of surgical debulking in patients with OMPC has always been considered impracticable, both for a marginal therapeutic role and for the greater risk of sequelae and/or complications related to the procedure itself. 

Several authors have demonstrated some mechanisms by which the persistence of the primary tumor can facilitate the clinical progression of the disease itself. Vassiliki et al^5^ demonstrate that despite 1 year of systemic therapies, with clinical-biochemical and radiological response, active clones of cells with high metastatic potential can be found within the primary tumor site. Equally, Kiera et al^6^ report that molecular signals of primary tumor may promote metastatic disease spread by altering the translation and regulation pathways of cellular signal. This results in cell proliferation, de-differentiation, cancer lymphatic and/or hematogenous dissemination, and angiogenesis.

Cifuentes et al^7^ first reported how surgical debulking of resectable PCas limits metastatic progression in a mouse model.

From these studies emerges the hypothesis of a possible therapeutic advantage in oncological terms also for cytoreductive radical prostatectomy (CRP), in a multimodal therapy setting, in patients with OMPC compared to systemic therapy alone.

### Definition End Prevalence of OMPC

No consensus exists to identify the OMPC. Actually, it is commonly defined by the presence of 5 or fewer metastatic sites on usual staging imaging

The prevalence of OMPC varies significantly in the literature. It strongly depends on the imaging examinations adopted to stage the disease. 

Same authors demonstrated a restaging of a cohort of patients initially diagnosed as locally confined PCa as OMPC patients by using higher sensitive imaging examinations such as 18-F-fluciclovine positron emission tomography (PET) scan. Likewise, patients diagnosed initially with OMPC were restaged as high volume metastatic disease using more sensitive imaging techniques.^8^ From this evidence emerges the need to make some considerations to frame the disease in a correct clinical and biological context, even before evaluating the feasibility of CRP in terms of safety and oncological results. First, the disease should be distinguished from the more aggressive one early diagnosed with a high polymetastatic potential. Biologically, OMPC should be considered a slow-growing and low-metastatic power disease. Support for this definition comes from the TROG 03.04 Randomised Androgen Deprivation and Radiotherapy (RADAR) trial (a phase III randomized study that compared 6 vs. 18 months of adjuvant ADT with or without zoledronic acid in men with intermediate/high-risk PCa undergoing radiotherapy (RT)). The study confirmed that patients with 4 or more metastasis had significantly higher PCa-specific mortality than those with oligometastatic disease (*P* = .004).^9^

Also, a distinction between de novo oligometastatic and recurrent oligometastatic disease should be made since their biological and clinical behavior is different.

**Safety, Feasibility, and Quality of Life of CRP in OMPC Patients**

There seems to be a consensus on the safety and the feasibility of CRP in OMPC patients and this evidence should encourage further investigation on surgical treatment (**Table 1** summarizes this evidence).

Heidenreich et al^10^ in a case–control study investigated the feasibility of CRP in patient with OMPC. No differences were reported in terms of frequency and seriousness of surgery-related complications in OMPC patients compared to high-risk non-metastatic PCa patients. 

Sooriakumaran et al^11^ in a multi-institutional analysis examined the safety of CRP in 106 patients with OMPC. No complication were experienced in 79.2% of patients. Positive-margin (53.8%), lymphocele (8.5%), and wound infection (4.7%) rates were higher than in surgery performed for locally confined disease. At 22.8 months follow-up, 88.7% of men were still alive. They concluded that surgery is safe in expert hands and that the overall and specific complication rates related to CRP were not higher than surgical treatment of locally confined PCa.

Conversely, Gandaglia et al^12^ in a single-center study, enrolling 11 patients, reported 20% of Clavien–Dindo grade-III complications, a significant increase in intraoperative blood loss requiring transfusion, and an increase in post-operative hospitalization compared to those undergoing surgical treatment for localized disease. They concluded that CRP has a tolerable safety profile and it is technically more challenging. 

Preisser et al.^13^ in a retrospective analyses conducted within the National Inpatient Sample database (2008-2013) including 874 OMPC patients compared open versus laparoscopic robotically assisted CRP. They observed significantly higher complications in the open surgery arm than in the laparoscopic robotically assisted one (overall complications: 10.0% vs. 21.4%, *P* = .001; blood transfusions: 2.6% vs. 11.2%, *P* = .001; miscellaneous medical: 4.1% vs. 8.3%, *P* = .01; and miscellaneous surgical complications, 2.2% vs. 4.9%, *P* = .046).

Chaloupka et al^14^ in a recent retrospective comparative study evaluated the effect of CRP on postoperative health-related quality of life (HRQOL). They found no significant difference in good general HRQOL rates between oligometastatic patients and localized disease patients before CRP (45.6% vs. 55.2%, *P* = .186) and during the follow-up (44% vs. 56%, *P* = .811). Global health status worsened significantly in localized disease patients compared to baseline (*P* = .001), whereas it did not change significantly in oligometastatic patients (*P* = .381). They concluded that there is no significant difference in HRQOL in OMPC patient after CRP, when compared to patients with organ-confined disease at the time of surgery.

### Oncological Outcomes of CRP in OMPC Patients

Looking at other metastatic diseases, there are deep evidence showing the benefit of cytoreductive surgery in terms of patient survival.^15^ In contrast, the role of CRP has not been rigorously evaluated. 

There are no studies able to answer this question, but prospective trials are ongoing [TRoMbone trial,^16^ SWOG S1802 randomized phase III trial,^17^ G-RAMMP phase III trial.^18^

Although the evidence is limited, there are some retrospective data suggesting a potential role for CRP in OMPC (**Table 2** summarizes these data).

To our knowledge, Austenfeld et al.^19^ in 1990, first reported significant benefits of CRP in well-selected OMPC patients in terms of progression rates or disease-free survival.

In a population-based study using the Munich Cancer Registry, Engel et al^20^ compared the overall mortality (OM) and relative survival of OMPC patients undergoing CRP (n = 688) and in patients who have not received local treatment (n = 250). An improvement in 10-year OM (64% vs. 28%) and relative survival (86% vs. 40%) was reported in the CRP group. Moreover, on multivariate analysis, CRP was an independent predictor of increased survival (*P* < .0001).

Heidenreich et al^10^ in their monocentric case–control study reported that the median time to castration-resistant disease was significantly higher in neoadjuvant ADT plus CRP patients (40 months, range 9-65) compared to ADT-alone treated patients (29 months, range 16-59) (*P* = .04). Patients in CRP group showed a better clinical progression-free survival (38.6 vs. 26.5 months, *P* = .032) and cancer-specific survival rates (95.6% vs. 84.2%, *P* = .043), whereas overall survival (OS) was similar. Moreover, one-third of patients who have not received local treatment required surgical treatment later for local cancer complications, such as obstruction, hematuria, and hydronephrosis.

Also, a recent review confirmed that CRP may be a useful choice in patients with severe local symptoms or oligometastatic disease.^21^

Parikh et al^22^ using the National Cancer Database (NCDB) evaluated the effects of local therapy (CRP, intensity-modulated radiation therapy [IMRT], or 2-dimensional(D)/3D conformal radiation therapy [CRT]) among patients with metastatic PCa diagnosed from 2004 to 2013. The study enrolled 6051 patients. No local therapy (LT) was administered in 5224 patients, while 622 (10.3%), 52 (0.9%), 153 (2.5%) patients respectively underwent CRP, IMRT, and 2D/3D CRT. Use of LT was associated with better health and oncological conditions (i.e., younger age (≤70), lower co-morbidity score, lower T-stage, Gleason score <8, node-negative status). Five-year OS was higher in patients receiving CRP than for those who have not received LT ( 45.7% vs. 17.1%, p < 0.01). In multivariate analysis, CRP and IMRT were independently associated with better OS (*P* < .01). 

Culp et al^23^ in a SEER-based retrospective study collected data of more than 8000 patients with all M-stage disease at diagnosis, in which 245 of these patients received CRP. The 5-year OS was greater (76.5% vs. 30.6%) and cancer-specific mortality rates were lower in CRP patients compared to no LT group. Other factors (age > 70, high-grade and T4 disease, Prostate Specific Antigen (PSA) ≥ 20 ng/mL, and pelvic lymphadenopathy) had independent association with cancer-specific mortality. The association with these factors was confirmed by Samuel et al^24^ in similar SEER-based retrospective study.

Löppenberg et al.,^25^ in an NCDB retrospective , compared CRP and no LT in terms of overall mortality (OM) in patients with all M stage PCa. Patients who received CRP had better 3-year OM free survival rates (63% vs. 48%; *P* < .001). Patients who yielded greatest benefit from surgical treatment were those with oligometastsatic disease and the lowest comorbidities/predicted OM risk. 

More recently, Steuber et al.^26^ in a monocentric prospective study, enrolled 43 OMPC patients undergoing CRP (median follow-up, 32.7 months) and 40 patients receiving standard systemic therapy only (median follow-up, 82.2 months). Inclusion criteria were de novo asymptomatic OMPC without visceral metastases, resectable disease, PSA < 150 ng/mL, no prior radiation of metastases, ADT synchronous therapy for both patients groups. No significant difference was reported in terms of castration resistant-free survival (*P* = .92) or OS (*P* = .25). However, CRP patients benefit from a significant reduction of local complications (7.0% vs. 35%; *P* < .01). To explain their contradictory results, authors evoked the selection bias of previous retrospective studies. 

## Conclusions

Oligometastatic prostate cancer is a transitional disease including a large spectrum of conditions with a polymorphic behavior and the correct diagnosis of OMPC requires more sensitive imaging techniques.

Cytoreductive radical prostatectomy is safe, feasible, and well-tolerated in a well-selected group of patients and, despite its challenging execution, it is associated with reduction of pelvic complications related to local disease progression. 

Treatment options are rapidly evolving and, waiting for the results of ongoing prospective-multicentric and randomized trials, a growing body of evidence suggests that a group of OMPC patients may receive deep and durable oncological benefits from CRP within a multimodality setting. 

## Statement of human rights

All procedures performed in studies involving human participants were in accordance with the ethical standards of the institutional and/or national research committee and with the 1964 Helsinki declaration and its later amendments or comparable ethical standards.

## Figures and Tables

**Table 1.** Summary of Studies Reporting Safety, Feasibility, and Quality of Life of OMPC Patients Who Underwent CRP

**Study**	**Time**	**Location**	**N**	**Results**
Heidenreich et al^10^	2015	Germany	23	Frequency and seriousness of surgery complications were not greater in OMPC patients than in patients with high-risk non-metastatic prostate cancer.
Sooriakumaran et al^11^	2016	Multicentric	106	Complication rates related to CRP were not more frequent than in radical prostatectomy for standard indications (79.2% of patients did not suffer any complications) Positive-margin (53.8%), lymphocele (8.5%), and wound infection (4.7%) rates were higher than open radical prostatectomy for standard indications 88.7% men were still alive at 22.8-month follow-up
Gandaglia et al^12^	2017	Italy	11	20% of Clavien–Dindo grade-III complications Significant increase of intraoperative blood loss requiring transfusion Increase in post-operative hospitalization
Preisser et al^13^	2019	USA	874 412 Retropubic Radical Prostatectomy (RRP) versus 462 Robot Assisted Laparoscopic Prostatectomy (RALP)	Overall complications (10.0% vs. 21.4%, *P* = .001),Blood transfusions complication (2.6% vs. 11.2%, *P* = .001), Miscellaneous medical (4.1% vs. 8.3%, *P* = .01) Miscellaneous surgical complications (2.2% vs. 4.9%, *P* = .046) significantly higher in RRP than in RALP.
Chaloupka et al^14^	2021	Germany		No significant difference in HRQOL in OMPC patient after CRP, when compared to patients with localized disease at time of surgery
TRoMbone	2017	UK	50	Waiting for results

OMPC, oligometastatic prostate cancer; CRP, cytoreductive radical prostatectomy; HRQOL, health-related quality of life.

**Table 2.** Summary of Studies Reporting CRP Oncological Outcomes

**Study**	**Type of Study**	**Time**	**Location**	**N**	**Results**
Engel et al^20^	Population based	2010	Germany	688 CRP versus 250 no LT	Increased 10-year OS (64% vs. 28%) and relative survival (86% vs. 40%) in the CRP group
Heidenreich et al^10^	Monocentric Case–control study	2015	Germany	23 CRP versus 38 no LT	Castration resistant prostate cancer of 40 versus 29 months (*P* = .04). Clinical progression-free survival 38.6 versus 26.5 months, (*P* = .032) CSS rates 95.6% versus 84.2%, (*P* = .043) Similar OS One-third of no LT patients require subsequent intervention for complications related to local progression (i.e., obstruction, hematuria, and hydronephrosis).
Parikh et al^22^	NCDB-based study	2017	USA	Total 6051 5224 no LT versus 827 LT (622 CRP + 52 IMRT + 153 2D/3D-CRT)	Five-year OS 45.7% versus 17.1% (*P* < .01). CRP (*P* < .01) and IMRT (*P* < .01) were independently associated with higher overall survival. After PS-matching, the use of LT remained significantly associated with overall survival (*P* < .01).
Culp et al^23^	SEER-based study	2014	USA	Total 8185 245 CRP + 129 BT versus 7811 no LT	5-year OS 76.5 in CRP group and 30.6 in no LT. Cancer-specific mortality rate was decreased in patients treated with CRP. CRP was associated with decreased cancer-specific mortality at all M stages Other factors had independent association with cancer-specific mortality (age > 70, high-grade and T4 disease, PSA ≥ 20 ng/mL, and pelvic lymphadenopathy).
Löppenberg et al^25^	NCDB-based study	2017	USA	Total 15 501 1470 CRP/RT versus 15 031 no LT	Three-year OM free survival rates (63% versus 48%; *P* < .001). Patients that yielded the greatest benefit from primary treatment were those with the lowest predicted OM risk, favorable disease burden, and little to no comorbidities. Patients with predicted OM risk greater than 70% had no survival benefit from local treatment.
Steuber et al^26^	Monocentric prospective	2017	Germany	43 CRP versus 40 no LT	No significant difference in castration resistant-free survival (*P* = .92) or OS (*P* = .25) was reported in this study. CRP patients benefit from a significant reduction in locoregional complications (*P* < .01)
SWOG S1802 randomized phase III trial^17^		Ongoing	USA	1273 Randomized	Waiting for results Primary outcome: overall survival
G-RAMMP phase III trial^18^		Ongoing	Germany	452 Randomized	Waiting for results Primary outcome: cancer-specific survival

CRP, cytoreductive radical prostatectomy; OS, overall survival; LT, laser therapy; OM, overall mortality; IMRT, intensity-modulated radiation therapy.
